# Supplementary material for: Programmatic Cost-Effectiveness of a Second-Time Visit to Detect New Tuberculosis and Diabetes Mellitus in TB Contact Tracing in Myanmar
Source: Int J Environ Res Public Health. 2022 Dec 1;19(23):16090. doi: 10.3390/ijerph192316090 (PMC9740873; doi:10.3390/ijerph192316090)
Supplement: Supplementary file 1 [file ijerph-19-16090-s001.zip › ijerph-2019883-supplementary.pdf]

Additional files

SUPPLEMENTARY TABLES

Table S1 Activities in contact tracing according to Myanmar National Tuberculosis Programme guideline, World Health Organization (WHO), and our current research

**Activities**	**Contact tracing (NTP guideline)**	**WHO Recommendation**	**Our current research**
First home visit			
- Signs and symptoms screening	+	+	+
- RBG test	+	+	+
- Provide health education	+	+	+
- Counselling to go to township TB clinic for:	Nil	All contacts	All contacts
o CXR	Only for SS (+) ve	Nil	Nil
o Sputum smear	Nil	Nil	+
o FBG test for contacts ≥ 25 years old			
Tests taken in township TB clinic			
- FBG test for contacts ≥ 25 years old	Nil	Nil	Contacts ≥ 25 years old
- CXR	For smear (-) ve but SS (+) ve	+	+
- GeneXpert test	For newly diagnosed TB	+	+

Activities	Contact tracing (NTP guideline)	WHO Recommendation	Our current research
Second home visit for household contacts did not come to TB clinic within two weeks			
- FBG test for contacts ≥ 25 years old	Nil	Nil	+
- Repeated health education	Nil	Nil	+
- Repeated counselling to take CXR in township TB clinic	Nil	Nil	+
Diagnosis of TB			
Bacterial confirmation			
- Sputum smear	+	Nil	Nil
- GeneXpert	Nil	+	+
CXR positive not cleared by two weeks of antibiotics among those who could not produce sputum (or) GeneXpert negative	+	+	+

NTP National Tuberculosis Programme, *RBG* random blood glucose test, *TB* tuberculosis, *CXR* chest X-ray, *SS* sputum smear, *FBG* fasting blood glucose test

Table S2 Cost estimates for capital and overhead costs

Item	Total number of tests (T)	Cost (USD) (C)	Annualizing factor (A)	Cost per test (USD) (C/(T×A))
**CXR examination**				
Capital cost				
Building space	9261 CXRs were taken in 2018	37,550	25.73 (50 years of useful life)	0.16
Digital CXR with portable generator	9261 CXRs were taken in 2018	115,523	8.53 (10 years of useful life)	1.46
Overhead cost				
Maintenance for quality control	9261 CXRs were taken in 2018	1,653	-	0.179
Electricity	9261 CXRs were taken in 2018	550	-	0.059
**GeneXpert test**				
Capital cost				
Building space	2075 tests in 2018	6,328	25.73 (50 years of useful life)	0.118
GeneXpert module with computer	2075 tests in 2018	18,740	8.53 (10 years of useful life)	1.059
Overhead cost				
Calibration	2075 tests in 2018	550	-	0.265
Electricity	2075 tests in 2018	550	-	0.265
**Glucometer**				
Overhead cost	617 tests	316.26	-	0.513

USD US dollars, *CXR* chest X-ray,

Table S3 Model input probabilities for parameters used in Monte Carlo simulation

Parameter	Base case	Distribution	Confidence interval	
			Low	High
**Compliance to screening after home visit**				
Full compliance to screening after 1st home visit	42.6%	Beta	31.8%	54.8%
Partial compliance to screening after 1st home visit	3.3%	Beta	0.1%	25.0%
Full compliance to screening after 2nd home visit	65.0%	Beta	53.2%	75.3%
Partial compliance to screening after 2nd home visit	18.2%	Beta	8.9%	29.9%
**Cost (USD)**				
Cost for first home visit	1.240	Gamma	1.076	1.432
Cost for second home visit	1.273	Gamma	1.117	1.437
Cost for RBG/FBG test	1.103	Gamma	0.974	1.256
Cost for chest X-ray	4.781	Gamma	3.462	6.376
Cost for GeneXpert	15.038	Gamma	8.709	22.511
**DALY averted**				
TB	1.800	Gamma	1.531	2.146
DM	0.038	Gamma	0.037	0.039

USD US dollars, *RBG* random blood glucose test, *FBG* fasting blood glucose test, *DALY* disability-adjusted life years, *TB* tuberculosis, *DM* diabetes mellitus

Table S4 Comparison of total costs between screening of TB and DM after first home visit versus second home visit

Activity	Quantity	Time spent in hours per household contacts	Cost per hour (USD)	Cost per quantity (USD)	Total cost for first visit (USD)	Total cost for second home visit (USD)	Total cost for two home visits (USD)
**Training for volunteers**							
Per diem for volunteers	8	≠	≠	3.77	30.12	≠	30.12
Per diem for trainers	2	≠	≠	11.30	22.59	≠	22.59
Refreshers	10	≠	≠	3.77	37.65	≠	37.65
Travel cost	10	≠	≠	1.13	11.30	≠	11.30
Printing	10	≠	≠	0.75	7.53	≠	7.53
**First home visit**							
Telephone call	193	0.05	1.13	0.06	10.89	≠	10.89
Home visit for contact tracing by volunteer	193	2	1.03	2.06	397.58	≠	397.58
Travel cost for volunteer	193	≠	≠	1.13	218.00	≠	218.00
Masks	519	≠	≠	0.04	28.05	≠	28.05
**Home-based RBG test**							
Glucometer	313	≠	≠	0.64	200.93	≠	200.93
Glucose strip	313	≠	≠	0.38	117.85	≠	117.85

Activity	Quantity	Time spent in hours per household contacts	Cost per hour (USD)	Cost per quantity (USD)	Total cost for first visit (USD)	Total cost for second home visit (USD)	Total cost for two home visits (USD)
Alcohol swab	313	≠	≠	0.02	5.66	≠	5.66
Needle	313	≠	≠	0.04	11.78	≠	11.78
**FBG test in TB clinic after 1st home visit**							
Duration of taking FBG test	127	0.07	1.03	0.07	8.76	≠	8.76
Glucometer	127	≠	≠	0.64	81.53	≠	81.53
Glucose strip	127	≠	≠	0.38	47.82	≠	47.82
Alcohol swab	127	≠	≠	0.02	2.30	≠	2.30
Needle	127	≠	≠	0.04	4.78	≠	4.78
**CXR examination after 1st home visit**							
Building	231	≠	≠	0.16	36.96	≠	36.96
Digital CXR with portable generator	231	≠	≠	1.46	337.26	≠	337.26
Maintenance for quality control	231	≠	≠	0.18	41.35	≠	41.35
Electricity	231	≠	≠	0.06	13.63	≠	13.63
CXR technician	231	0.56	1.20	0.67	155.23	≠	155.23
CXR film	231	≠	≠	1.24	286.44	≠	286.44
Radiologist	231	0.08	1.50	0.12	28.76	≠	28.76
Mask	231	≠	≠	0.04	8.70	≠	8.70

Activity	Quantity	Time spent in hours per household contacts	Cost per hour (USD)	Cost per quantity (USD)	Total cost for first visit (USD)	Total cost for second home visit (USD)	Total cost for two home visits (USD)
Materials use	231	≠	≠	0.85	196.35	≠	196.35
**Recheck CXR after 1st home visit**							
Building	50	≠	≠	0.16	8.00	≠	8.00
Digital CXR with portable generator	50	≠	≠	1.46	73.00	≠	73.00
Maintenance for quality control	50	≠	≠	0.18	8.95	≠	8.95
Electricity	50	≠	≠	0.06	2.95	≠	2.95
CXR technician	50	0.56	1.20	0.67	33.60	≠	33.60
CXR film	50	≠	≠	1.24	62.00	≠	62.00
Antibiotic	50	≠	≠	4.50	225.00	≠	225.00
Mask	50	≠	≠	0.04	1.88	≠	1.88
Radiologist	50	0.08	1.50	0.12	6.23	≠	6.23
**GeneXpert testing after 1st home visit**							
Building space	32	≠	≠	0.12	3.78	≠	3.78
GeneXpert module with computer	32	≠	≠	1.06	33.89	≠	33.89
Calibration	32	≠	≠	0.27	8.48	≠	8.48
Electricity	32	≠	≠	0.27	8.48	≠	8.48
Medical technologist	32	1.85	1.08	2.00	63.94	≠	63.94

Activity	Quantity	Time spent in hours per household contacts	Cost per hour (USD)	Cost per quantity (USD)	Total cost for first visit (USD)	Total cost for second home visit (USD)	Total cost for two home visits (USD)
Xpert testing	32	≠	≠	11.33	362.56	≠	362.56
Mask	32	≠	≠	0.04	1.20	≠	1.20
**HIV test in TB patients after 1st home visit**							
Test kit	15	≠	≠	1.12	16.80	≠	16.80
Duration of taking test	15	0.25	1.03	0.26	3.86	≠	3.86
Needles	15	≠	≠	0.04	0.56	≠	0.56
Alcohol swab	15	≠	≠	0.02	0.27	≠	0.27
**Additional RBG/FBG for ascertainment of undetermined DM after two times test in clinic**							
Glucometer	5	≠	≠	0.64	3.21	≠	3.21
Glucose strip	5	≠	≠	0.38	1.88	≠	1.88
Alcohol swab	5	≠	≠	0.02	0.09	≠	0.09
Needle	5	≠	≠	0.04	0.19	≠	0.19
Duration of taking RBG/FBG test	5	0.07	1.03	0.07	0.35	≠	0.35
**Second home visit**							
Telephone call	124	0.05	1.13	0.06	≠	7.00	7.00

Activity	Quantity	Time spent in hours per household contacts	Cost per hour (USD)	Cost per quantity (USD)	Total cost for first visit (USD)	Total cost for second home visit (USD)	Total cost for two home visits (USD)
Home visit for contact tracing by volunteer	124	1.75	1.03	1.80	≠	223.51	223.51
Travel cost for volunteer	124	≠	≠	1.13	≠	140.06	140.06
Mask	288	≠	≠	0.04	≠	15.55	15.55
**FBG test during second home visit**							
Glucometer	167	≠	≠	0.64	≠	107.21	107.21
Glucose strip	167	≠	≠	0.38	≠	62.88	62.88
Alcohol swab	167	≠	≠	0.02	≠	3.02	3.02
Needle	167	≠	≠	0.04	≠	6.29	6.29
**CXR examination after second home visit**							
Building	184	≠	≠	0.16	≠	29.44	29.44
Digital CXR with portable generator	184	≠	≠	1.46	≠	268.64	268.64
Maintenance for quality control	184	≠	≠	0.18	≠	32.94	32.94
Electricity	184	≠	≠	0.06	≠	10.86	10.86
CXR technician	184	0.56	1.20	0.67	≠	123.65	123.65
CXR film	184	≠	≠	1.24	≠	228.16	228.16

Activity	Quantity	Time spent in hours per household contacts	Cost per hour (USD)	Cost per quantity (USD)	Total cost for first visit (USD)	Total cost for second home visit (USD)	Total cost for two home visits (USD)
Masks	184	≠	≠	0.04	≠	6.93	6.93
Radiologist	184	0.08	1.50	0.12	≠	22.91	22.91
Material used in 2nd visit to TB clinic	184	≠	≠	0.85	≠	156.40	156.40
**Recheck CXR after second home visit**							
Building	31	≠	≠	0.16	≠	4.96	4.96
Digital CXR with portable generator	31	≠	≠	1.46	≠	45.26	45.26
Maintenance for quality control	31	≠	≠	0.18	≠	5.55	5.55
Electricity	31	≠	≠	0.06	≠	1.83	1.83
CXR technician	31	0.56	1.20	0.67	≠	20.83	20.83
CXR film	31	≠	≠	1.24	≠	38.44	38.44
Antibiotic	31	≠	≠	4.50	≠	139.50	139.50
Masks	31	≠	≠	0.04	≠	1.17	1.17
Radiologist	31	0.08	1.50	0.12	≠	3.86	3.86
**GeneXpert testing after second home visit**							
Building space	17	≠	≠	0.12	≠	2.01	2.01
GeneXpert module with computer	17	≠	≠	1.06	≠	18.00	18.00

Activity	Quantity	Time spent in hours per household contacts	Cost per hour (USD)	Cost per quantity (USD)	Total cost for first visit (USD)	Total cost for second home visit (USD)	Total cost for two home visits (USD)
Calibration	17	≠	≠	0.27	≠	4.51	4.51
Electricity	17	≠	≠	0.27	≠	4.51	4.51
Medical technologist	17	1.85	1.08	2.00	≠	33.97	33.97
Xpert testing	17	≠	≠	11.33	≠	192.61	192.61
Masks	17	≠	≠	0.04	≠	0.64	0.64
**HIV test after second home visit**							
Test kit	5	≠	≠	1.12	≠	5.60	5.60
Duration of taking test	5	0.25	1.03	0.26	≠	1.29	1.29
Needles	5	≠	≠	0.04	≠	0.19	0.19
Alcohol swab	5	≠	≠	0.02	≠	0.09	0.09
**Additional home visit for RBG/FBG test to ascertainment of undetermined DM**							
Home visit for contact tracing by volunteer	5	1.50	1.03	0.52	≠	7.73	7.73
Travel cost for volunteer	5	≠	≠	1.13	≠	5.65	5.65
Glucometer	5	≠	≠	0.64	≠	3.21	3.21
Glucose strip	5	≠	≠	0.38	≠	1.88	1.88
Alcohol swab	5	≠	≠	0.02	≠	0.09	0.09

Activity	Quantity	Time spent in hours per household contacts	Cost per hour (USD)	Cost per quantity (USD)	Total cost for first visit (USD)	Total cost for second home visit (USD)	Total cost for two home visits (USD)
Needle	5	≠	≠	0.04	≠	0.19	0.19
**Total cost**					**3280.95**	**1989.02**	**5269.97**

TB tuberculosis, *DM* diabetes mellitus, *USD* US dollars, *RBG* random blood glucose, *FBG* fasting blood glucose, *CXR* chest X-ray, *HIV* human immunodeficiency virus, ≠ not applicable

SUPPLEMENTARY FIGURE

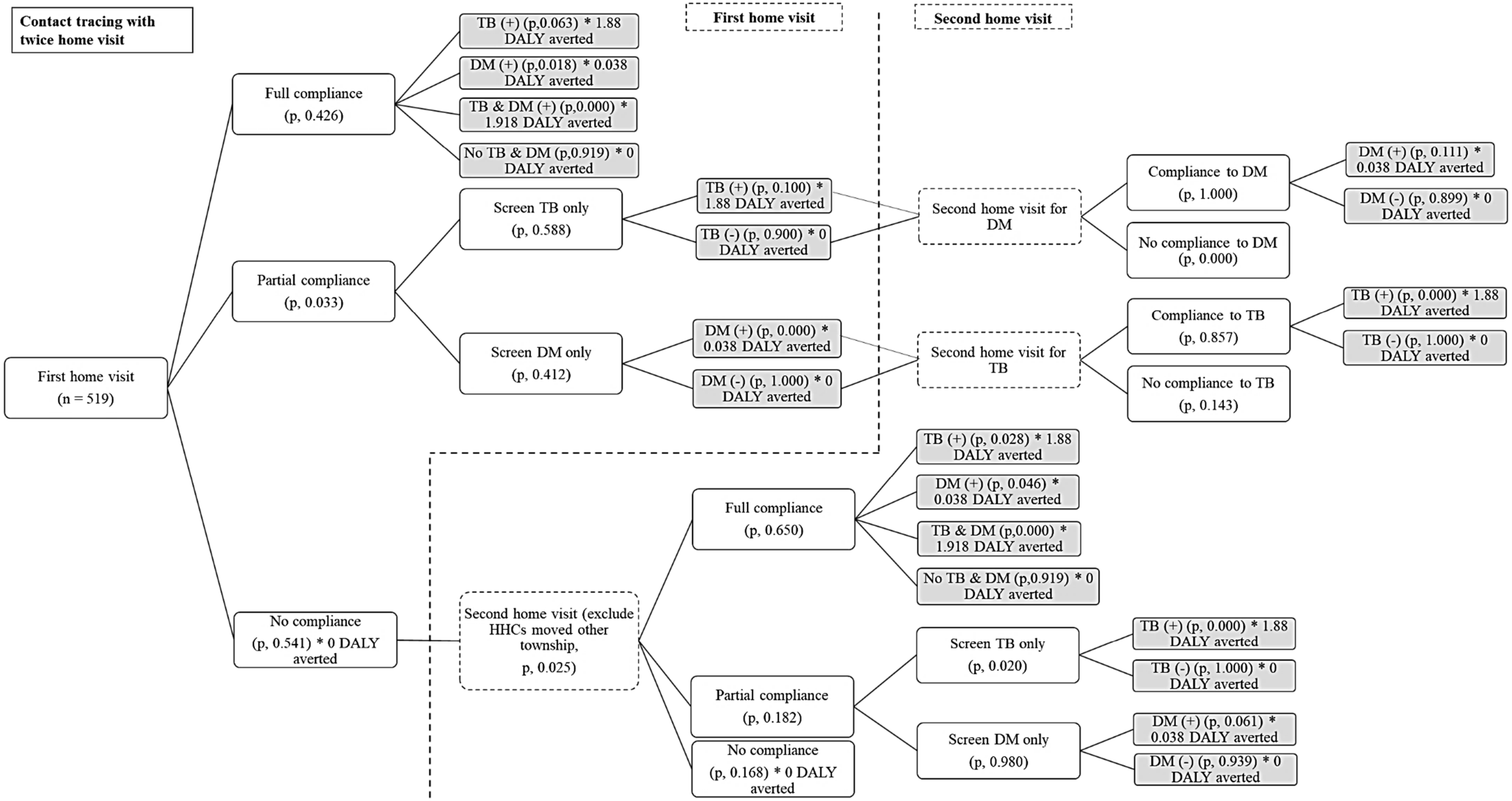

Figure S1 Decision tree of contact tracing for the first and second home visits. *TB* tuberculosis, *DM* diabetes mellitus, *p* probability, *DALY* disability-adjusted life years
